# Diagnostic, Treatment, and System Challenges in the Management of Recurrent Neuroleptic Malignant Syndrome on a General Medical Service

**DOI:** 10.1155/2018/4016087

**Published:** 2018-06-11

**Authors:** Karan Verma, Vivek Jayadeva, Raymond Serrano, Karthik Sivashanker

**Affiliations:** ^1^Department of Psychiatry, Boston University School of Medicine, Boston Medical Center, Boston, MA, USA; ^2^VA Boston Healthcare System, Boston, MA, USA; ^3^VA Boston Healthcare System, Brockton Division, Harvard South Shore Psychiatry Residency, Brockton, MA, USA; ^4^Brigham and Women's Hospital, Boston, MA, USA

## Abstract

Neuroleptic malignant syndrome (NMS), an iatrogenic form of malignant catatonia, carries high morbidity and mortality rates especially in the context of delayed recognition and standard intervention protocol of lorazepam trial. However, there is limited guidance available through literature for further management if benzodiazepine treatment is ineffective and electroconvulsive therapy (ECT) is not readily accessible. This case report describes a multimodal approach to address the diagnostic, treatment, and logistical system challenges in an acute medical hospital through the case of a 69-year-old man with schizophrenia who represented from a psychiatric ward with neuroleptic malignant syndrome. We educated our inpatient colleagues for timely recognition of hyperexcited subtype of catatonia to avoid iatrogenic progression to neuroleptic malignant syndrome and our medical colleagues on the clinical course of catatonic symptoms to avoid any further disagreements and delays in treatment. We advocated for timely electroconvulsive therapy in the setting of limited access and utilized creative pharmacologic strategies such as N-methyl-D-aspartate (NMDA) receptor antagonists and longer acting benzodiazepines while managing medical complications.

## 1. Introduction

Neuroleptic malignant syndrome (NMS) has been conceptualized as an iatrogenic form of malignant catatonia secondary to antipsychotic use (see Table 1) [1]. It is characterized by a clinical tetrad of fever, lead-pipe rigidity, autonomic instability, and altered mental status [1–6]. Other extrapyramidal signs (EPS) such as cogwheeling, coarse tremors, akinesia, or dystonia may occur before autonomic symptoms. The evolution of symptoms over one to three days may be accompanied by elevated creatine kinase (typically more than 1000 IU/L), leukocytosis (10,000 to 40,000 cells/mm3 with a left shift), impaired liver functioning, electrolyte derangements, and renal impairment secondary to myoglobinuria [4, 5]. If not recognized and managed promptly, this syndrome carries a high potential for morbidity and mortality [1, 3, 7–12]. Discontinuation of the offending agent and supportive care remain the first-line interventions [10–14]. Due to shared pathomechanisms of motor abnormalities with catatonia, intravenous lorazepam is gold standard treatment. In lorazepam nonresponders or severe cases requiring rapid intervention, electroconvulsive therapy (ECT) is the definite intervention, with a reported response rate of 85% [15]. The literature provides limited guidance to clinicians regarding further management, when standard interventions fail or are unavailable. We describe a case of neuroleptic malignant syndrome with refractory catatonia complicated by delirium, provider disagreement regarding the diagnosis, and a delay in timely electroconvulsive therapy (ECT) that required creative solutions to achieve clinical improvement.

## 2. Case Presentation

A 69-year-old Caucasian man with schizophrenia represented to our emergency department (ED) from a psychiatric hospital with catatonia, notable for agitation and altered mental status requiring physical restraints. Limited physical exam was revealing for increased tone and rigidity in bilateral lower extremities while the patient self-dialogued and yelled at times. Per outside records, he was observed to be persistently agitated, engaging in self-injurious behaviors such as hitting himself, banging his head, and refusing to eat or drink for a week.

Three weeks prior, he was admitted to the medicine service with early signs of NMS that resolved over the course of a few days with discontinuation of neuroleptics and treatment with parenteral lorazepam. He was subsequently transferred back to the outside hospital psychiatric unit for further stabilization and optimization of his psychotropic regimen, with a recommendation to avoid high-potency neuroleptics. There, he was started on fluphenazine, a high-potency first generation antipsychotic, after a washout period of one week. His religious delusions with disorganized thought process showed minimal improvement. He was subsequently switched to haloperidol, which was rapidly increased to 35 mg per day. Clonazepam 1.5 mg per day and lorazepam 1 mg per day were also utilized over this time frame. The patient, however, became increasingly agitated, with self-injurious behavior and some posturing that was attributed to “refractory psychosis.” This prompted further antipsychotic dose escalation. He had stopped eating or drinking by this time with associated worsening of behavioral dysregulation. 75 mg of chlorpromazine was given the same day after 35 mg of haloperidol showed minimal benefit. While chlorpromazine temporarily decreased his behavioral dysregulation, his agitation continued unabated the following morning. He was given additional chlorpromazine 25 mg with fluid resuscitation in urgent care before his transfer to our facility for a delirium work-up.

On arrival, standard treatment was implemented, including antipsychotic discontinuation, supportive care, and initiation of parenteral benzodiazepines consisting of lorazepam 2 mg intravenously (IV) every 8 hours. His complete blood count, electrolytes, vitamin B12, thyroid function, and liver function tests were within normal limits, except for a CK of 1090 IU/L, mildly elevated 10,680 white blood cells per mcL, low iron level of 26 ug/dL, and creatinine value of 1.26 mg/dL thought to be prerenal in nature secondary to dehydration. He tested negative for syphilis on the rapid plasma reagin test. Urinalysis did not reveal an infection. Urine drug toxicology was positive for benzodiazepines only, which the patient had been receiving on the inpatient unit. A head CT revealed no acute intracranial pathology. An electroencephalogram performed on day 4 of admission was notable only for increased beta waves, reflecting the high-dose benzodiazepines he was receiving at the time.

The patient's hospital course was notable for continued fluctuating motor symptoms with episodes of severe rigidity in both upper and lower extremities, intermittent droning vocalizations, waxy flexibility, posturing, negativism, automatic obedience, and presence of mitgehen. Such periods were accompanied by diaphoresis and autonomic hyperactivity. On the fifth day, lorazepam was increased to 4 mg IV every 4 hours and amantadine 100 mg (by mouth) PO twice a day was started the following day. The doses were held past midnight due to planned ECT on the seventh day of admission. Lorazepam was resumed post-ECT but amantadine was held until after the third session. Prior to each subsequent ECT treatment, benzodiazepines were held to prevent increasing the patient's seizure threshold. He received a total of three ECT sessions, each three days apart. In between the second and the third ECT sessions, the patient became overtly delirious secondary to a urinary tract infection, which was treated with antibiotics. After the second ECT session, lorazepam was decreased to 2 mg IV every 6 hours and then switched to a longer acting diazepam 20 mg IV every 6 hours after the third ECT session. Amantadine was restarted at the same dose and was increased to 100 mg PO three times a day, four days after the last ECT session. After a three-week washout period, Clozapine 12.5 mg PO twice a day was introduced for underlying psychotic symptoms and titrated to 25 mg twice a day without recurrence of NMS or catatonia. With gradual lysing of catatonia and normalization of lab values, the patient was discharged back to the inpatient psychiatric unit after a month-long hospitalization.

## 3. Discussion

NMS may be difficult to distinguish from a variety of medical and psychiatric conditions. Medically, NMS may appear like nonconvulsive status epilepticus, severe Parkinson's disease, locked-in syndrome, or akinetic mutism from brain injury [13]. Psychiatrically, NMS may be mistaken for extrapyramidal symptom, such as antipsychotic-induced Parkinsonism. NMS is associated with both first- and second-generation antipsychotic use (SGAs), though SGAs are associated with lower incidence and clinical severity [7, 16].

Most commonly, NMS can be indistinguishable from malignant catatonia (the most severe form of catatonia), except for the precipitating factor of antipsychotic treatment as noted in Table 1. Malignant catatonia and NMS are often conceptualized on the same spectrum and some have even proposed them as “two variants of the same disorder” due to dysfunctional central dopaminergic systems that account for motor symptoms [4, 9, 12]. In addition to history, there are certain clinical features that may assist with the diagnostic process. Positive motoric findings, such as dystonic posturing, waxy flexibility, and stereotyped movements, are seen more commonly in malignant catatonia than NMS [16]. As with our patient, a prominent behavioral or affective prodrome marked by agitation or catatonic excitement is also more common in malignant catatonia than NMS [16]. In this case, this catatonic prodrome was misattributed to “refractory psychosis,” prompting escalating antipsychotic use, which further exacerbated catatonic symptoms and ultimately resulted in NMS. More timely recognition of the catatonic prodrome may have avoided further clinical deterioration to a less treatment responsive state. Of note, nosologic confusion and poor operationalization has contributed to high variability in reported prevalence rates [17]. That said, although the incidence of catatonia appears to have declined over the decades, underrecognition of catatonia remains an issue. A recent systemic review found the incidence of catatonia to be up to 18% in psychiatric inpatients and up to 30% of patients with delirium [18]. Our case further adds to the published notion of this under recognition, especially the excited subtype of this syndrome, with an opportunity for refresher education for inpatient mental health providers.

A similar theme of misdiagnosis emerged when the patient was assessed by the neurology service after he had received lorazepam for 5 days. Due to his fluctuating clinical course even within a day and interrater variability, his Bush Francis Catatonia Rating Scale (BFCRS) scores differed significantly on their exam from ours. They attributed his akinesia and cogwheeling to neuroleptic-induced Parkinsonism rather than to residual symptoms of resolving NMS (see Table 1). Therefore, they suggested withholding amantadine, an NMDA antagonist with some dopaminergic activity. We educated the primary medical team regarding the intermittent nature of muscle rigidity and the full range of observable movements in catatonia, as found by Castillo and colleagues in their literature review [3]. We recommended amantadine as a temporary treatment bridge while awaiting ECT equipment. This recommendation was based on emerging evidence implicating a dysfunctional glutamatergic system in catatonia. It has been hypothesized that the lack of glutamate inhibition due to massive GABA depletion in the supplementary motor areas results in a net effect of glutamatergic-mediated excitation in the striatum [4, 12, 19]. Carroll and colleagues recommended initiating an NMDA antagonist when catatonia is unresponsive to benzodiazepines or ECT [20]. We suggested that the addition of an NMDA antagonist may be useful, while awaiting coordination of ECT. It is challenging to directly ascribe any clinical improvement to amantadine, given its relatively brief and interrupted use in this case, in combination with other therapies. That said, it was well tolerated with no adverse effects and appeared to provide a delayed response 1-7 days after initiation, as described in the literature [20].

**Figure 1:**
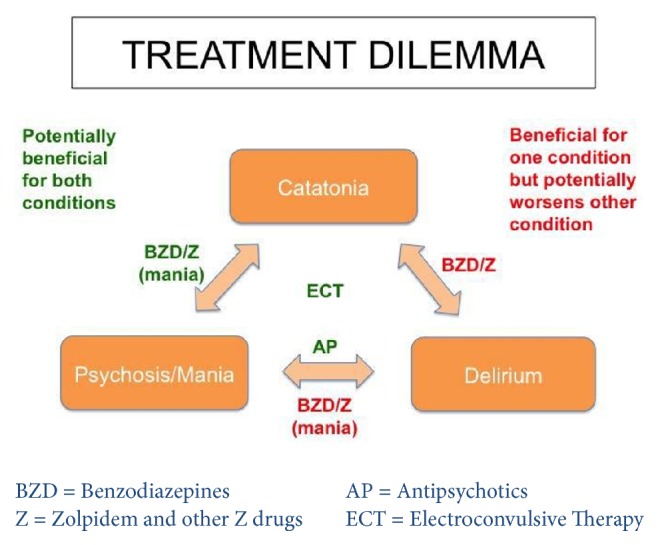


Although DSM-5 states that catatonia should not be diagnosed if it occurs in the context of a delirium, symptoms of catatonia and delirium commonly overlap (see Table 1) [16], antipsychotic agents, which are typically first-line treatments for hyperactive delirium, may worsen catatonia or precipitate NMS, as seen with our patient. Conversely, benzodiazepines are the first-line treatment for catatonia but may worsen delirium (see Table 1) [17]. Given this clinical dilemma (see Figure 1), NMDA antagonists may be particularly useful in catatonic patients with concurrent delirium [17]. Another etiology for his encephalopathic presentation especially in the context of significant urine retention was thought to be chlorpromazine-induced anticholinergic syndrome. However, features such as diaphoresis, rigidity, and elevated CK levels are not typical for this syndrome.

ECT is effective in relieving catatonia after failure of high doses of IV within the first 72 hours [20–22]. In our patient with clinical disagreements across providers, this had to be further delayed due to the lack of a readily available ECT machine in our acute medical hospital. Our service involved psychiatry leadership to request their assistance in expediting ECT for this patient given his tenuous clinical state and potential for high morbidity and mortality. Some case reports have found reduced treatment response due to delayed use of ECT [17]. This case highlights the need for the structural capacity and clinical expertise to administer ECT emergently in the acute medical setting.

We also utilized benzodiazepines in a novel manner, given the aforementioned logistical challenges and suboptimal clinical response to lorazepam. We hypothesized that troughs and peaks in his serum level of lorazepam could contribute to fluctuations in his clinical presentation over the course of a day. Therefore, we switched to a longer acting benzodiazepine—diazepam—to ensure a steadier serum level, which appeared to be helpful in this case. Further studies are ultimately needed to determine if there are clinically meaningful differences between benzodiazepines for catatonia treatment. In the absence of rigorous comparative trials between benzodiazepines, it is reasonable to assume that they share a class effect via GABA-A modulation for treatment of catatonia, similar to treatment of alcohol withdrawal. Therefore, we would suggest that pharmacodynamic and pharmacokinetic factors, such as half-life, should be a stronger consideration when selecting a benzodiazepine for catatonia treatment. As the most studied benzodiazepine in treatment of catatonia, lorazepam remains the gold standard, but a switch to a longer acting benzodiazepine may be considered for lorazepam partial responders or those with a highly fluctuant course.

Of note, we did not administer dantrolene as mentioned in Table 1. Although some cases have reported success with dantrolene treatment, Reulbach and colleagues suggested that it may not be the evidence-based treatment of choice in cases of NMS [23]. They found that combination treatment with dantrolene prolonged the complete time of remission, and, with monotherapy, mortality was increased, though patients were more severely ill in this group. Pileggi and Cook note in a recent review that dantrolene's utility is typically reserved for severely ill patients [24]. Our patient's clinical course was tenuous with slow resolution but not severe enough to require the intensive care unit level of care.

After a 3-week washout period, clozapine was chosen for its mesolimbic selectivity and initiated with no recurrence of NMS or catatonia. The recommendation to titrate dose for psychotic remission was made to our inpatient psychiatric colleagues.

## 4. Conclusion

This case highlights the diagnostic and treatment dilemmas in the management of general medicine service patients admitted with conditions such as NMS, catatonia, and delirium. A multimodal approach that empowers medical colleagues through education addresses barriers to timely ECT for general medicine patients, utilizes creative pharmacologic strategies such as NMDA antagonists and longer acting benzodiazepines, and manages concurrent conditions such as delirium may be helpful to patients experiencing a combination of NMS, catatonia, and delirium in the general medical setting.

## Figures and Tables

**Table 1:** Differential diagnosis.

	NMS	Malignant Catatonia	Neuroleptic-induced Parkinsonism	Delirium
Key features	Autonomic Instability, Delirium, Fever, Rigidity; Precipitated by use of an antipsychotic	Autonomic Instability, Delirium, Fever, Rigidity	Postural and resting tremors, Rigidity (Cogwheel), Oral-buccal dyskinesias (concurrent)	Waxing and waning consciousness, Inattention, Perceptual deficits, Behavioral disorganization

Notable Lab Values	Elevated serum CK, leukocytosis, electrolyte abnormalities, low serum iron	Typically normal	Typically normal	Dependent on etiology

Treatment	Dantrolene, Bromocriptine, Benzodiazepines, NMDA antagonists, ECT	Benzodiazepines, ECT, NMDA antagonists	Stopping the offending agent; if not, Anticholinergics, Amantadine	Typically antipsychotics; benzodiazepines for GABAergic withdrawal or status epilepticus
